# Association between TGF-β1 -913G/C polymorphism and myocardial infarction risk in a Chinese Han population: a case–control study

**DOI:** 10.1042/BSR20190315

**Published:** 2019-06-20

**Authors:** Lili Wu, Guofan Chen, Jia Song

**Affiliations:** 1 Department of Nursing, Sir Run Run Shaw Hospital, Zhejiang University School of Medicine, 3 Qingchun East Road, Hangzhou, Zhejiang, China; 2 Department of Cardiology, The Affiliated Hospital of Hangzhou Normal University, 126 Wenzhou Road, Hangzhou, Zhejiang, China; 3 Department of Emergency, the Second Affiliated Hospital of Zhejiang Chinese Medical University, 318 Chaowang Road, Hangzhou, Zhejiang, China

**Keywords:** case-control study, myocardial infarction, polymorphism, TGF-β1

## Abstract

Transforming growth factor (TGF)-β1 contributed to angiotensin II (Ang II)-mediated collagen accumulation after myocardial infarction (MI). The present study aimed to investigate the association of genetic variant of TGF-β1 gene with the risk of MI. The present study recruited a total of 530 MI patients and 651 healthy controls. The genomic DNA was extracted and subjected into polymerase chain reaction (PCR) and Sanger sequencing. The present study indicated that TGF-β1 -913G/C polymorphism was associated with increased risk for MI under the co-dominant, dominant and allelic models. The increased risk effect was also evident among the females, younger subjects (age < 60 years), smokers, non-drinkers and individuals with hypertension. Additionally, the present study observed significant differences among cases and controls in terms of total cholesterol (TC). In conclusion, TGF-β1 -913G/C polymorphism is associated with increased risk for MI. TGF-β1 -913G/C polymorphism may be a potential prognostic biomarker for MI.

## Introduction

Myocardial infarction (MI), a clinical complication of coronary atherosclerosis, primarily develops from the rupture of an atherosclerotic plaque with thrombus formation and the occlusion of the coronary vessel [1], thereby leading to a sharp reduction in the blood supply to the myocardium [2,3]. MI causes more than 2.4 million deaths in the America, and more than 4 million deaths were reported in Europe and northern Asia [4]. Approximately 550000 first episodes and 200000 recurrent episodes of MI occur annually [5]. Risk factors including hypertension, diabetes mellitus, smoking, hyperlipidemia and obesity were reported to be associated with increased risk for MI [6,7]; however, the underlying mechanisms resulting in MI are still unclear. MI, a complex multifactorial disorder, is affected by both environmental factors and genetic predisposition. Functional gene variants have been reported to affect the susceptibility to MI [8,9].

Transforming growth factor (TGF)-β1 is a cytokine expressed in platelets, hematopoietic, connective and endothelial tissue cells [10]. TGF-β1 is the most common isoform of the TGF-β family, which regulates cell growth, differentiation and matrix production [11]. TGF-β1 could increase the mRNA expressions of endothelin in vascular endothelial cells [12] and the synthesis of extracellular matrix components such as collagen [13]. TGF-β1 gene is located on chromosome 19q13.2 and a single nucleotide polymorphism (SNP) was identified in exon 1 at position -913G/C (rs1800471) of the TGF-β1 gene. TGF-β1 -913G/C polymorphism was associated with TGF-β1 protein production in peripheral blood leukocytes [14]. Several studies [15–17] have explored the relationship between TGF-β1 rs1800471 polymorphism and MI risk; however, they yielded conflicting findings. A German study found -913G/C polymorphism was not associated with the risk of MI [16], while other studies showed -913G/C polymorphism was related to increased risk for MI [15,17]. Up to date, no studies from China investigated the relationship between TGF-β1 -913G/C polymorphism and MI susceptibility. Thus, we conducted this case–control study to investigate the association between TGF-β1 -913G/C polymorphism and the risk of MI.

## Materials and methods

### Study subjects

The present study of a total of 530 patients diagnosed with MI and 651 gender- and age-matched controls were recruited from the Affiliated Hospital of Hangzhou Normal University, and the Second Affiliated Hospital of Zhejiang Chinese Medical University from April 2013 to April 2018. The typical ECG changes, elevated cardiac markers and clinical history were used for diagnosis. Coronary angiography was used to identify the responsible stenosis in any of the major coronary arteries or in the left main trunk. Patients with history of coronary artery disease, renal or hepatic disease, cardiomyopathy and malignant tumor were excluded from case groups.

The control groups were recruited from individuals who had physical examinations in our hospital. Controls with sinus rhythm (ECG data) and normal heart function (UCG data) in the normal condition were enrolled in this case–control study. The controls excluded individuals with a history of atherosclerosis, rheumatic heart disease and liver, and kidney dysfunction by medical history. A standard questionnaire was used to obtain demographic of all participants, including smoking status, drinking status, history of MI, hypertension and diabetes mellitus. All MI patients and healthy controls were interviewed and recorded about demographic and risk factors. Individuals were defined as smoker if they smoked at least one cigarette per day for more than 1 year. Individuals who drank three alcoholic drinks in a week. If the systolic/diastolic blood pressure exceeded 140/90 mmHg [18,19] or they self-reported history of hypertension, they were described as hypertensive. Diabetes mellitus was diagnosed if fasting glucose levels were higher than 110 md/dl or 2-h glucose levels ≥ 11.1 mmol/l in an oral glucose, which was recommended by World Health Organization (WHO) [20–22]. The data of total cholesterol (TC), high-density lipoprotein (HDL), low-density lipoprotein (LDL) and triglyceride (TG) were obtained from medical records.

We obtained written informed consent from all subjects. All procedure has been reviewed in line with Ethical Standards of the Affiliated Hospital of Hangzhou Normal University, and the Second Affiliated Hospital of Zhejiang Chinese Medical University. The present study was in accordance with the Helsinki declaration.

### DNA extraction and genotyping

Peripheral blood samples (2 ml) were collected in tubes containing EDTA for genotype analysis. Genomic DNA was extracted using the TIANamp Blood DNA kit (Tiangen Biotech, Beijing, China) in accordance with manufacturer’s instructions. TGF-β1 gene -913G/C polymorphism was genotyped by using polymerase chain reaction (PCR) and Sanger sequencing. The primers were 5′-TGGCATGAACCGGCCTT-3′ (forward) and 5′-GCCGGGAGCTTTGCAGAT-3′ (reverse). PCR was performed under the following conditions: an initial holding at 95°C for 10 min, 40 cycles of denaturation at 92°C for 15 s and annealing and extension at 60°C for 1 min, and a final holding at 60°C for 1 min. The PCR products were sequenced by using ABI 3500 Genetic Analyzer. The sequence data were analyzed by using Sequence Scanner Version 1.0. Further, 2% of samples were repeatedly genotyped for quality control.

### Statistical analysis

The differences of the demographic characteristics and risk factors between MI patients and controls were analyzed using chi-square test (for categorical variables) and Student’s test and one-way ANOVA (for continuous variables). Departure from the Hardy–Weinberg (HWE) among controls was evaluated using a goodness-of-fit chi-square test. The odds ratio (OR) and 95% confidence interval (CI) were calculated to evaluate the effect of TGF-β1 -913G/C polymorphism on the risk of MI by using logistic regression analysis adjusted for age and sex. *P*-value less than 0.05 was considered as statistically significant. All statistical analyses were conducted using SPSS 20.0 software (SPSS Inc, Chicago, U.S.A.).

## Results

### Characteristics of study subjects

The demographic characteristics of participants are shown in Table 1. The average age of cases and controls was 57.68 and 57.16 years respectively, which did not differ significantly. There was no significant difference among cases and controls in the distribution of sex and TG level (*P*>0.05). There were more smokers, alcohol consumers and individuals with hypertension or diabetes mellitus among the MI patients than those among the healthy controls. Additionally, the MI patients had higher levels of body mass index (BMI), TC, and LDL, but lower HDL.

**Table 1:** Patient demographics and risk factors in MI

Variable	Cases (*n*=530)	Controls (*n*=651)	*P*
Age (years)	57.68 ± 8.80	57.16 ± 9.31	0.327
Sex			0.122
Male	187 (35.3%)	202 (31.0%)	
Female	343 (64.7%)	449 (69.0%)	
BMI	26.83 ± 3.40	26.78 ± 3.45	0.812
Smoking			<**0.001**
Yes	301 (56.8%)	179 (27.5%)	
No	229 (43.2%)	472 (72.5%)	
Alcohol			<**0.001**
Yes	157 (29.6%)	124 (19.0%)	
No	373 (70.4%)	527 (81.0%)	
Hypertension			<**0.001**
Yes	333 (62.8%)	231 (35.5%)	
No	197 (37.2%)	420 (64.5%)	
Diabetes mellitus			<**0.001**
Yes	261 (49.2%)	119 (18.3%)	
No	269 (50.8%)	532 (81.7%)	
HDL	1.36 ± 0.28	1.33 ± 0.49	0.265
LDL	3.07 ± 0.94	2.64 ± 0.91	<**0.001**
TC	4.77 ± 1.22	4.66 ± 1.09	0.084
TG	0.20 ± 0.07	0.21 ± 0.08	0.973

### TGF-β1 -913G/C polymorphism and MI risk

The distribution of genotypes and alleles of the TGF-β1 -913G/C polymorphism in MI cases and controls are provided in Table 2. The observed genotypes distribution for this SNP was in line with HWE among the controls (*P*=0.546). Our results indicated that TT genotype (TT vs. CC; OR, 1.94; 95% CI, 1.01–3.72, *P*=0.048), but not CT genotype, had a significantly elevated risk for MI, compared with CC genotype. Similarly, CT+TT genotype had an increased susceptibility to MI (CT+TT vs. CC; OR, 1.32; 95% CI, 1.04–1.68, *P*=0.025). The association was also significant after adjusting sex and age. Additionally, TGF-β1 -913G/C polymorphism was also associated with increased risk for MI in the allelic model (T vs. C; OR, 1.31; 95% CI, 1.06–1.61, *P*=0.011).

**Table 2:** Logistic regression analysis of associations between TGF-β1 -913G/C polymorphisms and risk of MI

Genotype	Cases* (*n*=530)		Controls* (*n*=651)		OR (95% CI)	*P*	OR* (95% CI)	P*
	*n*	%	*n*	%				
rs1800471 C/T								
CC	332	62.8%	448	69.0%	1.00	-	-	-
CT	174	32.9%	185	28.5%	1.27 (0.99–1.63)	0.065	1.27 (0.99–1.64)	0.059
TT	23	4.3%	16	2.5%	**1.94 (1.01–3.72)**	**0.048**	**1.94 (1.01–3.74)**	**0.048**
CT+TT	197	37.2%	201	31.0%	**1.32 (1.04–1.68)**	**0.025**	**1.33 (1.04–1.69)**	**0.023**
CC+CT	506	95.7%	633	97.5%	1.00			
TT	23	4.3%	16	2.5%	1.80 (0.94–3.44)	0.077	1.80 (0.94–3.44)	0.077
C allele	838	79.2%	1081	83.3%	1.00	-	-	-
T allele	220	20.8%	217	16.7%	**1.31 (1.06–1.61)**	**0.011**	-	-

The genotyping was successful in 529 cases and 649 controls for TGF-β1 -913G/C polymorphism.

Bold values are statistically significant (*P*<0.05).

* Adjust sex and age.

### The relationship between TGF-β1 -913G/C polymorphism and clinical and biochemical characteristics of MI

Stratification analyses were conducted according to sex, age, smoking, alcohol, hypertension and diabetes mellitus. The increased risk associated with TT+CT genotypes was more pronounced among the females, smokers, individuals without hypertension (Table 3). This significant association also held true among non-drinkers and age < 60 years individuals. We further evaluated the effect of -913G/C polymorphism on the clinical parameters among the participants. The TC level differed significantly among the different genotypes of -913G/C polymorphism (*P*=0.001) (Table 4). Next, we observed no evidence of association between this SNP and other parameters (age, BMI, HDL, LDL and TG) (Table 4).

**Table 3:** Stratified analyses between TGF-β1 -913G/C polymorphism and the risk of MI

Variable	-913G/C (case/control)			CT vs. CC	TT vs.CC	TT vs. CT+CC	TT+CT vs.CC
	CC	CT	TT				
Sex							
Male	123/140	56/56	8/5	1.14 (0.73–1.77); 0.566	1.82 (0.58–5.71); 0.304	1.75 (0.56–5.45); 0.333	1.19 (0.78–1.83); 0.414
Female	209/308	118/129	15/11	1.34 (0.99–1.82); 0.058	2.00 (0.90–4.45); 0.088	1.82 (0.82–4.01); 0.139	**1.40 (1.04–1.88); 0.027**
Smoking							
Yes	191/130	97/44	12/5	1.49 (0.98–2.27); 0.064	1.62 (0.56–4.71); 0.375	1.44 (0.50–4.16); 0.499	**1.50 (1.00–2.25); 0.049**
No	141/318	77/141	11/11	1.23 (0.88-1.73); 0.232	2.26 (0.96-5.32); 0.064	2.11 (0.90-4.93); 0.086	1.31 (0.94-1.81); 0.112
Alcohol							
Yes	93/86	60/35	4/4	1.55 (0.93–2.58); 0.093	0.90 (0.22–3.73); 0.888	0.78 (0.19–3.17); 0.726	1.48 (0.90–2.43); 0.120
No	239/364	114/150	19/12	1.15 (0.86–1.55); 0.337	**2.41 (1.15–5.05); 0.020**	**2.30 (1.10–4.80); 0.026**	1.25 (0.94–1.65); 0.124
Hypertension							
Yes	214/157	103/65	15/7	1.16 (0.80–1.69); 0.428	1.57 (0.63–3.95); 0.336	1.50 (0.60–3.74); 0.384	1.20 (0.84–1.72); 0.313
No	118/291	71/120	8/9	**1.45 (1.01–2.09); 0.043**	2.19 (0.82–5.80); 0.117	1.93 (0.73–5.08); 0.184	**1.51 (1.06–2.14); 0.023**
Diabetes mellitus							
Yes	161/77	84/38	15/4	1.06 (0.66–1.69); 0.816	1.79 (0.58–5.59); 0.314	1.76 (0.57–5.42); 0.325	1.13 (0.72–1.77); 0.603
No	171/371	90/147	8/12	1.33 (0.96–1.82); 0.084	1.44 (0.58–3.59); 0.431	1.32 (0.53–3.27); 0.548	1.33 (0.98–1.82); 0.069
Age (years)							
<60	196/274	106/109	12/5	1.36 (0.98–1.87); 0.067	**3.34 (1.16–9.64); 0.026**	**3.04 (1.06–8.71); 0.039**	**1.44 (1.05–1.98); 0.023**
≥60	136/174	68/76	11/11	1.15 (0.77–1.70); 0.504	1.28 (0.54–3.04); 0.577	1.23 (0.52–2.89); 0.642	1.16 (0.80–1.70); 0.437

Bold values are statistically significant (*P*<0.05).

**Table 4:** The clinical and biochemical characteristics of TGF-β1 -913G/C polymorphism between two groups

	Patients (*n*=530)				Controls (*n*=651)			
	CC (*n*=332)	CT (*n*=174)	TT (*n*=23)	*P*	CC (*n*=296)	CT (*n*=151)	TT (*n*=16)	*P*
Age (years)	57.37 ± 8.86	57.67 ± 8.65	61.65 ± 7.79	0.077	56.93 ± 9.44	57.27 ± 8.93	60.69 ± 8.46	0.272
BMI (kg/m^2^)	26.91 ± 3.39	26.77 ± 3.38	26.27 ± 3.83	0.656	26.81 ± 3.50	26.72 ± 3.40	26.65 ± 2.95	0.947
TC (mmol/l)	6.63 ± 1.08	4.69 ± 1.08	5.27 ± 0.99	**0.024**	4.73 ± 1.26	4.81 ± 1.06	4.69 ± 0.68	0.733
HDL (mmol/l)	1.38 ± 0.28	1.33 ± 0.28	1.33 ± 0.28	0.194	1.33 ± 0.49	1.35 ± 0.48	1.21 ± 0.41	0.550
LDL (mmol/l)	3.09 ± 0.94	3.08 ± 0.95	2.79 ± 0.92	0.341	2.63 ± 0.91	2.64 ± 0.92	2.91 ± 0.80	0.496
TG (mmol/l)	0.21 ± 0.07	0.20 ± 0.06	0.21 ± 0.08	0.739	0.21 ± 0.07	0.20 ± 0.07	0.21 ± 0.06	0.900

The genotyping was successful in 529 cases and 649 controls for TGF-β1 -913G/C polymorphism.

## Discussion

In the present study, our data showed that TGF-β1 -913G/C polymorphism was associated with MI risk in this Chinese Han population. This increased effect was more evident among the females, younger subjects (age < 60 years), smokers, non-drinkers, and individuals without hypertension. Furthermore, this SNP was demonstrated to be associated with the TC level.

Koch et al. [16] first investigated the relationship between TGF-β1 -913G/C polymorphism and MI risk in a German population with 3657 MI patients and 1211 control individuals. They found -913G/C polymorphism was not associated with the risk of MI [16]. However, they indicated that -509C/868T/913G/11929C (CTGC) haplotype were associated with MI in men, but not in women [16], suggesting that sex was a risk factor for the interaction between this SNP and MI susceptibility. Next, an Iranian study found that -913G/C polymorphism conferred susceptibility to MI [17]. It was observed that the mRNA expression and circulating levels of TGF-β1 were significantly higher in the MI patients than those in control subjects [17]. In addition, they showed -913G/C polymorphism was related to the increased levels of TGF-β1 gene and protein in MI patients [17]. Barsova et al. [15] from Russia also explored the association between this SNP and MI risk in a population with 406 cases and 198 controls. They replicated the positive findings of the Iranian study [17], and showed -913G/C polymorphism was a risk factor for MI patients [15]. However, Chen et al. [23] from England revealed that TGF-β1 -913G/C polymorphism was not the risk of ischemic heart disease and MI in patients with rheumatoid arthritis.

In the present study, we found that TGF-β1 -913G/C polymorphism was related to increased risk for MI in a Chinese Han population. It is obvious that abovementioned studies [15–17] yielded conflicting findings regarding the relationship between -913G/C polymorphism and MI risk. Herein, we assumed the following factors may account for it. One, genetic heterogeneity for MI was shown in different races. Two, clinical heterogeneity was an important factor. The study from Iran [17] enrolled acute MI patients, while the German study [16] explored MI patients with angiographically proven coronary heart disease. Three, the sample sizes varied between these studies. Four, diverse exposure factors may also explain it.

Several limitations should be considered in the present study. One, the sample size of this case–control study was limited, which may make the findings of the present study underpowered. A multi-center case–control study with larger sample size is warranted in the future. Two, we only explored one SNP of TGF-β1 gene. Three, selection bias in this retrospective study should not be ignored. Four, further study uncovering the underlying mechanism why TGF-β1 gene polymorphism conferred susceptibility to MI was warranted. Five, the follow-up data were missing, which restricted further analysis about the relationship between this SNP and other clinical symptoms. Last, the interaction between genetic factors and environmental factors should be performed.

In conclusion, the present study finds that TGF-β1 -913G/C polymorphism is associated with increased risk for MI in a Chinese Han population. Other studies with larger sample sizes in Chinese Han population are urgently needed.

## Abbreviations

BMI: body mass index
CI: confidence interval
ECG: Electrocardiogram
HDL: high-density lipoprotein
HWE: Hardy–Weinberg
LDL: low-density lipoprotein
MI: myocardial infarction
OR: odds ratio
PCR: polymerase chain reaction
SNP: single nucleotide polymorphism
TC: total cholesterol
TG: triglyceride
TGF: transforming growth factor
UCG: Ultrasound Cardiogram
